# Acute ethanol exposure has bidirectional actions on the endogenous neuromodulator adenosine in rat hippocampus

**DOI:** 10.1111/bph.14152

**Published:** 2018-03-25

**Authors:** Victoria Hughes, Magnus J E Richardson, Mark J Wall

**Affiliations:** ^1^ School of Life Sciences University of Warwick Coventry UK; ^2^ Department of Mathematics University of Warwick Coventry UK

## Abstract

**Background and Purpose:**

Ethanol is a widely used recreational drug with complex effects on physiological and pathological brain function. In epileptic patients, the use of ethanol can modify seizure initiation and subsequent seizure activity with reports of ethanol being both pro‐ and anticonvulsant. One proposed target of ethanol's actions is the neuromodulator adenosine, which is released during epileptic seizures to feedback and inhibit the occurrence of subsequent seizures. Here, we investigated the actions of acute ethanol exposure on adenosine signalling in rat hippocampus.

**Experimental Approach:**

We have combined electrophysiology with direct measurements of extracellular adenosine using microelectrode biosensors in rat hippocampal slices.

**Key Results:**

We found that ethanol has bidirectional actions on adenosine signalling: depressant concentrations of ethanol (50 mM) increased the basal extracellular concentration of adenosine under baseline conditions, leading to the inhibition of synaptic transmission, but it inhibited adenosine release during evoked seizure activity in brain slices. The reduction in activity‐dependent adenosine release was in part produced by effects on NMDA receptors, although other mechanisms also appeared to be involved. Low concentrations of ethanol (10–15 mM) enhanced pathological network activity by selectively blocking activity‐dependent adenosine release.

**Conclusions and Implications:**

The complex dose‐dependent actions of ethanol on adenosine signalling could in part explain the mixture of pro‐convulsant and anticonvulsant actions of ethanol that have previously been reported.

**Abbreviations**
ADO: biosensor adenosine biosensor
ENT: equilibrative nucleoside transporter
INO: biosensor inosine biosensor

## Introduction

http://www.guidetopharmacology.org/GRAC/LigandDisplayForward?ligandId=2299 has complex effects on brain function, which are still not fully understood. In low doses, ethanol can be an excitant whereas in higher doses, it is a depressant (for review, see Hendler *et al.,* 2013). These contradictory effects have also been observed in patients with epilepsy, as ethanol can act as an anticonvulsant (Fischer, 2005) but can also be pro‐convulsant (Cohen *et al*., 1993; reviewed in Leach *et al.,* 2012) in particular leading to the relapse of patients who are stabilized by medication (Gordon and Devinsky, 2001). The mechanisms of ethanol's effect on network activity at the cellular and molecular level are still not fully characterized and may differ depending on brain region (see Harrison *et al*., 2017 for review). For a long period, ethanol was believed to exert its effects by producing a general depression. However, it has become increasingly clear that ethanol interacts with a number of specific neurotransmitter systems including http://www.guidetopharmacology.org/GRAC/LigandDisplayForward?ligandId=1067 (where it enhances the http://www.guidetopharmacology.org/GRAC/FamilyDisplayForward?familyId=72 conductance in a concentration‐dependent manner, Förstera *et al.,* 2016) and http://www.guidetopharmacology.org/GRAC/LigandDisplayForward?ligandId=1369 (Möykkynen and Korpi, 2012), interacts with specific voltage‐gated channels (e.g. http://www.guidetopharmacology.org/GRAC/FamilyDisplayForward?familyId=81, Bettinger and Davies, 2014; http://www.guidetopharmacology.org/GRAC/FamilyDisplayForward?familyId=80, Walter and Messing, 1999) and can also directly modify cell membrane function (Fleuret‐Balter *et al.,* 1983). All of these effects could potentially change the threshold for seizures and modify seizure duration.

It was postulated over 30 years ago that ethanol could produce some of its effects by interacting with http://www.guidetopharmacology.org/GRAC/LigandDisplayForward?ligandId=2844 signalling mechanisms (first suggested by Dar *et al.,* 1983, and reviewed in Mailliard and Diamond, 2004; Ruby *et al.,* 2010; Nam *et al.,* 2013). In particular, there is strong evidence that ethanol can increase the extracellular concentration of adenosine in the brain (Sharma *et al.,* 2010), and some of the effects of ethanol can be reduced by adenosine receptor antagonists (Franks *et al.,* 1975). Adenosine is a potent neuromodulator involved in many physiological and pathological processes (reviewed in Dunwiddie and Masino, 2001; Sebastião and Ribeiro, 2009; Borea *et al.,* 2016). Adenosine acts *via* multiple cell‐surface GPCRs, with the high‐affinity inhibitory http://www.guidetopharmacology.org/GRAC/ObjectDisplayForward?objectId=18 being the most widely expressed (reviewed in Fredholm *et al.,* 2000). Presynaptic A_1_ receptors inhibit the release of neurotransmitters (first discovered by Vizi and Knoll, 1976), and A_1_‐receptor activation also hyperpolarizes neuronal membranes. Adenosine can be released into the extracellular space by a number of mechanisms: directly *via* equilbrative nucleoside transporters (ENTs, Lovatt *et al.,* 2012; Wall and Dale, 2013) as http://www.guidetopharmacology.org/GRAC/LigandDisplayForward?ligandId=1713 from neurons (Pankratov *et al*., 2007) or glial cells (Newman, 2004; Pascual *et al.,* 2005; Wall and Dale, 2013) and then metabolized in the extracellular space, or adenosine can be released directly as a neurotransmitter (Klyuch *et al.,* 2012). During epileptic seizures, adenosine is released into the extracellular space to activate A_1_ receptors to terminate bursts of activity and to delay or prevent the onset of the next seizure (During and Spencer, 1992; Dale and Frenguelli, 2009; Wall and Richardson, 2015). In hippocampal and neocortical slices, adenosine release during epileptiform activity has been directly measured and characterized with microelectrode biosensors (Frenguelli and Wall, 2015; Wall and Richardson, 2015).

In the present study, we used a combination of electrophysiology and microelectrode biosensors to produce the first direct characterization of the effects of acute ethanol exposure on adenosine signalling during epileptiform activity. In most experiments, we used 50 mM ethanol, which is a concentration that can be measured in the blood stream of heavy drinkers (reviewed in Harrison *et al*., 2017). We found that ethanol has contradictory effects, enhancing the basal concentration of adenosine but also inhibiting the release of adenosine during seizure activity. These effects may help to explain the pro‐ and anticonvulsant effects of ethanol that have been reported previously.

## Methods

### Preparation of hippocampal slices

All animal care and experimental procedures were reviewed and approved by the institutional animal welfare and ethical review body (University of Warwick). Animal studies are reported in compliance with the ARRIVE guidelines (Kilkenny *et al*., 2010; McGrath and Lilley, 2015).

Sagittal slices of hippocampus (400 μm) were prepared from male Sprague Dawley rats, at postnatal days 20–30 (Wall and Dale, 2013). Rats were kept on a 12 h light–dark cycle with slices made from rats killed 90 min after entering the light cycle. In accordance with the U.K. Animals (Scientific Procedures) Act (1986), male rats were killed by cervical dislocation and decapitated. The brain was removed, cut down the mid line and the two sides of the brain stuck down to the base plate. Slices were cut around the midline with a Microm HM 650 V microslicer in cold (2–4°C) high Mg^2+^, low Ca^2+^ aCSF, composed of (mM): 127 NaCl, 1.9 KCl, 8 MgCl_2_, 0.5 CaCl_2_, 1.2 KH_2_PO_4_, 26 NaHCO_3_, 10 D‐glucose (pH 7.4 when bubbled with 95% O_2_ and 5% CO_2_, 300 mOsm). Slices were stored at 34°C for 1–6 h in aCSF (1 mM MgCl_2_, 2 mM CaCl_2_) before use.

### Extracellular and biosensor recording from hippocampal slices

A slice was transferred to the recording chamber, submerged in aCSF and perfused at 4–6 mL·min^−1^ (32°C); the slice was placed on a grid allowing perfusion above and below the tissue, and all tubing was gas tight (to prevent loss of oxygen). For extracellular recording, an aCSF filled microelectrode was placed on the surface of stratum radiatum in CA1. Extracellular recordings were made using a differential model 3000 amplifier (AM systems, WA, USA) with field EPSPs (fEPSPs) and adenosine release evoked with an isolated pulse stimulator model 2100 (AM Systems, WA). For fEPSPs, a 10–20 min baseline was recorded at a stimulus intensity that gave 40–50% of the maximal response. Signals were filtered at 3 kHz and digitized on line (10 kHz) with a Micro CED (Mark 2) interface controlled by Spike software (*Vs* 6.1, Cambridge Electronic Design, Cambridge, UK). For fEPSP slope, a 1 ms linear region after the fibre volley was measured. Standard cylindrical microelectrode biosensors were inserted into the slice, so that biosensors went through the slice in stratum radiatum in area CA1 (Wall and Dale, 2013). Slices were then allowed to recover before measurements were made.

### Biosensor characteristics

Biosensors (Sarissa Biomedical Ltd, Coventry, UK) consist of enzymes trapped within a matrix around a Pt or Pt/Ir (90/10) wire (Llaudet *et al.,* 2003). Biosensors were cylindrical with an exposed length of ~500 μm and diameter of ~50 μm. Three types of sensor were used in this study: firstly, null sensors, possessing the matrix but no enzymes, to control for non‐specific electro‐active interferents; secondly, biosensors containing http://www.guidetopharmacology.org/GRAC/ObjectDisplayForward?objectId=1230, http://www.guidetopharmacology.org/GRAC/ObjectDisplayForward?objectId=2841 and http://www.guidetopharmacology.org/GRAC/ObjectDisplayForward?objectId=2646&familyId=920&familyType=ENZYME (responsive to adenosine, inosine and hypoxanthine: ADO biosensors); and thirdly, biosensors containing nucleoside phosphorylase and xanthine oxidase (responsive to http://www.guidetopharmacology.org/GRAC/LigandDisplayForward?ligandId=4554 and http://www.guidetopharmacology.org/GRAC/LigandDisplayForward?ligandId=4555: INO biosensors).

A full description of biosensor properties has been published previously (Llaudet *et al.,* 2003); they show a linear response to increasing concentrations of analyte and have a fast response time (<1 s, Wall and Richardson, 2015). In each experiment, the biosensors were calibrated with the analyte (10 μM) before and after insertion in the slice, to check for loss of sensitivity. An application of 10 μM http://www.guidetopharmacology.org/GRAC/LigandDisplayForward?ligandId=5 was used to check that the screening layer was intact. In many of the experiments, the composition of purines detected by ADO biosensors was not fully defined. Since ADO biosensors have an equal sensitivity to adenosine, inosine and hypoxanthine (Llaudet *et al.,* 2003; Wall *et al.,* 2007), the total concentration of purines detected was related to the calibration to adenosine to give μM' or nM' of purines (as outlined in Pearson *et al.,* 2001; Klyuch *et al.,* 2011). For simplicity, we refer to biosensor measurements as adenosine rather than purines. Biosensor signals were acquired at 1 kHz with a Micro CED (Mark 2) interface using Spike (*Vs* 6.1) software.

### Measuring the effects of ethanol on adenosine release during seizure activity

To determine the effects that acute ethanol exposure has on adenosine release during seizure activity, seizure activity was induced in hippocampal slices with zero Mg^2+^ aCSF with 50 μM http://www.guidetopharmacology.org/GRAC/LigandDisplayForward?ligandId=2416) (reviewed in Frenguelli and Wall, 2015). Adenosine release was monitored with an adenosine biosensor (with the signal on the null sensor subtracted). In most experiments, ethanol was applied at a concentration of 50 mM. Experiments were done in two ways: firstly, slices were pre‐incubated in ethanol (10–15 min) and then perfused with zero Mg^2+^ aCSF and 50 μm 4‐AP (ethanol still present), and responses were compared with interleaved slices where no ethanol was applied. Secondly, seizure activity was established, and then ethanol was applied.

### Deconvolution and reconvolution of purine waveforms

The amplitude of closely spaced waveforms produced by the release of adenosine is difficult to quantify accurately as subsequent pulses sit on the decay and overlap with preceding ones. Following Richardson and Silberberg (2008), closely spaced release‐events were deconvolved as in Klyuch *et al*. (2011) by removing the long decay τ_o_ component. The resulting sharper, well‐spaced events were then cropped and reconvolved (using the same decay constant) to yield isolated waveforms from which the amplitude and rise time could be accurately and straight forwardly measured (Wall and Richardson, 2015; Frenguelli and Wall, 2015). All analyses was done in the JULIA programming environment.

### Statistical methods

All values are given as mean ± SEM. Statistical significance was tested with one‐way ANOVA and Student's paired or unpaired *t*‐tests. The data and statistical analysis comply with the recommendations on experimental design and analysis in pharmacology (Curtis *et al*., 2015).

### Drugs

Drugs were made up as stock solutions (1–10 mM) and then diluted in aCSF on the day of use. Drugs used: the http://www.guidetopharmacology.org/GRAC/FamilyDisplayForward?familyId=75 antagonist http://www.guidetopharmacology.org/GRAC/LigandDisplayForward?ligandId=4239 (Tocris), the A_1_ receptor antagonist http://www.guidetopharmacology.org/GRAC/LigandDisplayForward?ligandId=385) (Sigma), the equilibrative nucleoside transporter inhibitors http://www.guidetopharmacology.org/GRAC/LigandDisplayForward?ligandId=4512 and http://www.guidetopharmacology.org/GRAC/LigandDisplayForward?ligandId=4807 (Sigma) and the non‐selective glutamate receptor antagonist http://www.guidetopharmacology.org/GRAC/LigandDisplayForward?ligandId=2918 (Tocris). Ethanol was directly added to the aCSF just before application to the slice.

### Nomenclature of targets and ligands

Key protein targets and ligands in this article are hyperlinked to corresponding entries in http://www.guidetopharmacology.org, the common portal for data from the IUPHAR/BPS Guide to PHARMACOLOGY (Harding *et al.,* 2018), and are permanently archived in the Concise Guide to PHARMACOLOGY 2017/18 (Alexander *et al*., 2017a,b,c,d).

## Results

### Ethanol does not affect biosensor sensitivity to adenosine

**Figure 1:**
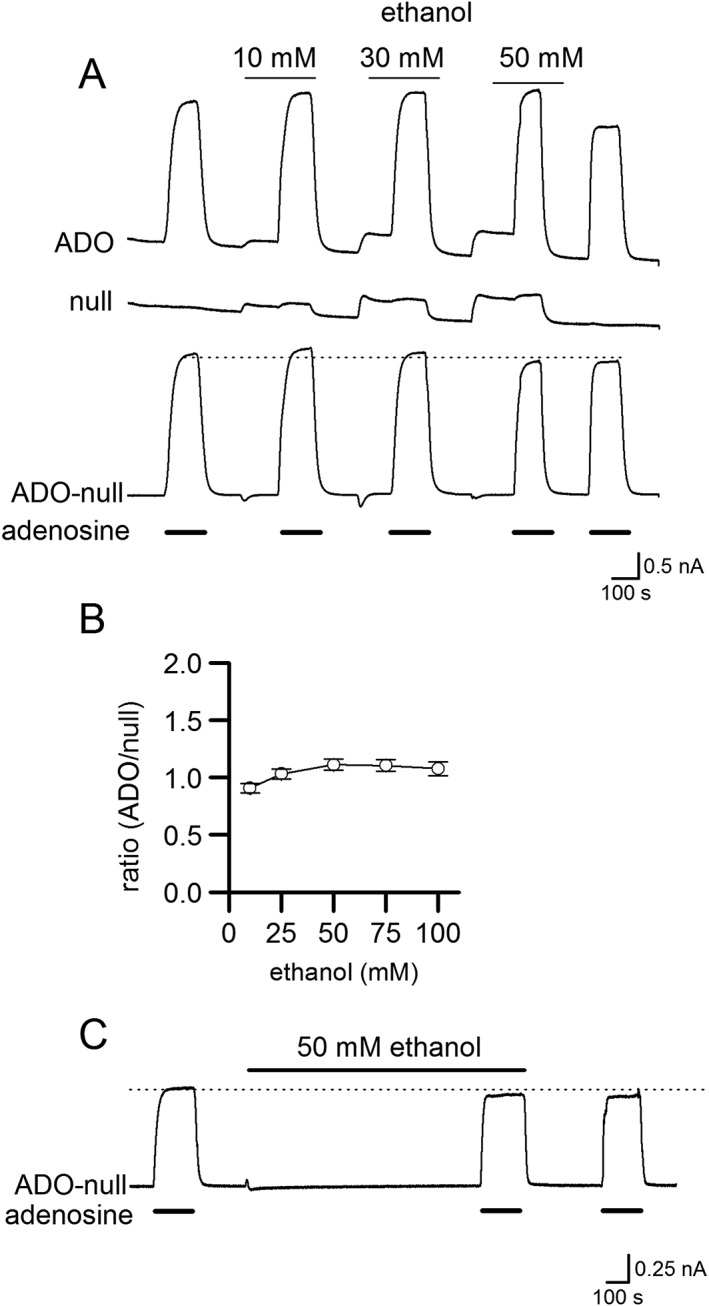
Effect of ethanol on biosensor properties. (A) Traces from an adenosine biosensor (ADO), null sensor and the ADO biosensor (null subtracted). Increasing concentrations of ethanol (10–50 mM) induced an upward shift in the baseline current on both null and ADO biosensor but had no effect on adenosine (10 μM) calibration currents. Subtraction of the null trace from the ADO biosensor trace removed the baseline shift. (B) Graph plotting the mean ratio of the ethanol‐induced current on ADO biosensor versus the null sensor against ethanol concentration (three ADO biosensor and null sensor pairs). (C) Trace from an ADO biosensor (null subtracted). Application of ethanol (50 mM for 25 min) had no effect on biosensor sensitivity to adenosine (10 μM).

We first tested whether ethanol has effects on biosensor properties that could impair adenosine measurements. With no tissue present, ethanol induced a concentration‐dependent (10–100 mM) positive deflection (100–300 pA) in the baseline current of the ADO biosensor (Figure 1A). Since this effect was of a similar magnitude on the null sensor (which lacks the detecting enzymes, Figure 1A), this suggests that ethanol produces a direct electrochemical effect on the polarized biosensor. By subtracting the current on the null sensor from the ADO biosensor current, the effects of ethanol on the baseline current could be removed (Figure 1A, B; *n* = 4). Ethanol (10–100 mM) had no significant effect on the sensitivity of the biosensor to adenosine (Figure 1A, *n* = 4). Long applications of ethanol (up to 30 min, 50 mM) also had no effect on biosensor sensitivity (*n* = 3, Figure 1C). Thus, ethanol does not change the sensitivity of the biosensor, and the linear increase in baseline current produced by ethanol can be removed by subtracting the signal on the null sensor.

### Ethanol can increase the basal extracellular adenosine concentration

**Figure 2:**
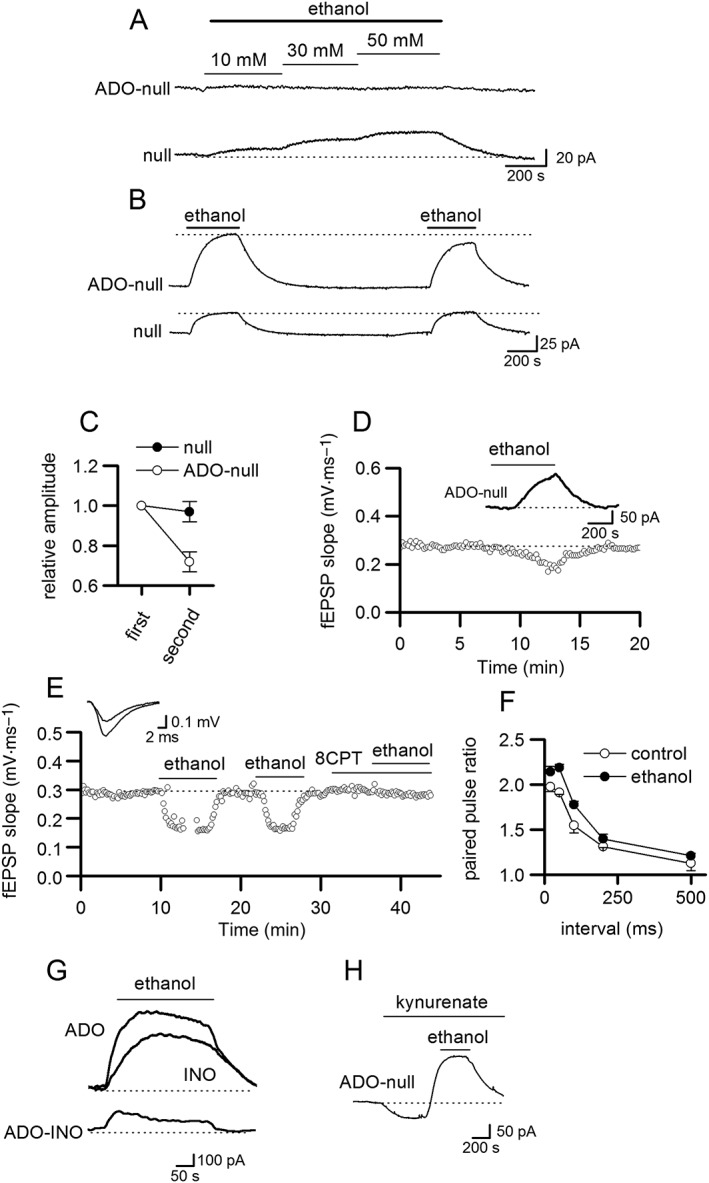
Ethanol increases extracellular adenosine concentration. (A) Example trace from an ADO biosensor (null subtracted) and null sensor that have been placed in area CA1. Ethanol induced a shift in ADO baseline current, which was removed by null subtraction. No net increase in the adenosine biosensor current was observed in 62% of slices. (B) Traces from an ADO biosensor (null subtracted) and null sensor. Ethanol (50 mM) induced an upward shift in the ADO baseline current that persisted after null subtraction. This was the case for 38% of slices. (C) Graph plotting the relative amplitude of currents induced by ethanol (50 mM) for first and second applications measured on the null sensor and the ADO biosensor (null subtracted). Currents were normalized to the amplitude of the current produced by the first application of ethanol. (D) Graph plotting fEPSP slope versus time for an individual slice. Ethanol (50 mM) reversibly decreased fEPSP slope. Inset, trace from an ADO biosensor (null subtracted). (E) Graph plotting fEPSP slope against time for an individual slice. The effect of ethanol (50 mM) was blocked by the A_1_ receptor antagonist 8CPT (2 μM). Inset, superimposed fEPSP averages in control and in ethanol. (F) Graph of paired pulse ratio against pulse interval in control and in 50 mM ethanol. Ethanol significantly increased the paired pulse ratio at short intervals (up to 100 ms but had no effect on intervals at 200 and 500 ms). (G) Superimposed current traces from an ADO and INO biosensor. Subtracting the scaled INO trace from ADO trace revealed an adenosine current in response to 50 mM ethanol. (H) Trace from an ADO biosensor (null subtracted). The glutamate receptor antagonist kynurenate (5 mM) did not prevent the ethanol (50 mM)‐induced current.

We first tested whether acute ethanol application changes the basal extracellular concentration of adenosine. An ADO biosensor and null sensor were inserted into CA1, and ethanol was applied. In the majority of slices (25 out of 40), the ethanol‐induced upward shift in baseline current was the same amplitude on the ADO biosensor as the null sensor (in four cases, the signal on the null was slightly greater than on the ADO biosensor), and thus, upon subtraction, there was no net increase in current (Figure 2A). Thus, in these slices, there is no detectable change in the extracellular concentration of adenosine when ethanol was applied. However, in 15 out of 40 hippocampal slices, 20–50 mM ethanol induced a net signal on the ADO biosensor: the current deflection on the ADO biosensor persisted after subtracting the null sensor signal (mean current after subtracting current on null sensor for 50 mM ethanol, 175 ± 30 pA, equivalent to ~0.8 μM' of adenosine, Figure 2B). This increase in baseline current on the ADO biosensor could be observed with concentrations of ethanol above 20 mM and increased in amplitude as the concentration of ethanol was increased (tested up to 50 mM). With repeated applications, the amplitude of the current on the ADO biosensor significantly diminished (*P* < 0.05 one‐way ANOVA), unlike the current on the null sensor that remained at a constant amplitude (Figure 2B, C) suggesting depletion of adenosine stores. To test whether sufficient adenosine was released to inhibit synaptic transmission, fEPSPs were recorded simultaneously with biosensor measurements. In the slices where there was a net ADO biosensor current, fEPSP slope was reduced (Figure 2D, *n* = 3) with a similar time course to the biosensor current. In slices in which there was no net ADO biosensor current, fEPSP slope was unaffected by ethanol application (not illustrated *n* = 3). In a further six slices, fEPSP was recorded without biosensor measurements, ethanol decreased the initial slope of fEPSPs (mean inhibition of fEPSPs with and without biosensor measurements 30.8 ± 4.3%, *n* = 9 slices). In the six slices, the inhibition could be reversed by blocking A_1_ receptors with the antagonist 8CPT (2 μM 8CPT increased fEPSP slope by 12 ± 3%, Figure 2E). The inhibition produced by ethanol significantly (*P* < 0.05, one‐way ANOVA) increased paired pulse facilitation (at short intervals 100 ms and less) consistent with a change in neurotransmitter release probability and the actions of adenosine at presynaptic A_1_ receptors (Figure 2F, Dunwiddie and Haas, 1985). Differential measurements with ADO and INO biosensors revealed a rapidly rising transient signal upon subtraction that is consistent with the direct detection of adenosine (Figure 2G, *n* = 4 out of 10 recordings, in the other six recordings, no clear adenosine signal was discernible). This adenosine component diminished with repeated ethanol applications.

Adenosine can be released by the activation of ionotropic glutamate receptors (Wall and Dale, 2013). However, the increase in extracellular adenosine concentration produced by ethanol was not dependent on glutamate receptors as it persisted in the presence of 5 mM kynurenate, a non‐selective antagonist at NMDA and http://www.guidetopharmacology.org/GRAC/FamilyDisplayForward?familyId=75 receptors (Figure 2H, *n* = 5). Another possible mechanism is inhibition of adenosine transporters. This does not seem likely as the effects of ethanol were very rapid, unlike the effects of blocking transporters (e.g. see Dunwiddie and Diao, 1994; Frenguelli *et al.,* 2007). Furthermore, the effects of ethanol persisted in the presence of ENT inhibitors (NBTI and dipyridamole *n* = 5, not illustrated).

### Ethanol can reduce the basal continuous A_1_ receptor activation

**Figure 3:**
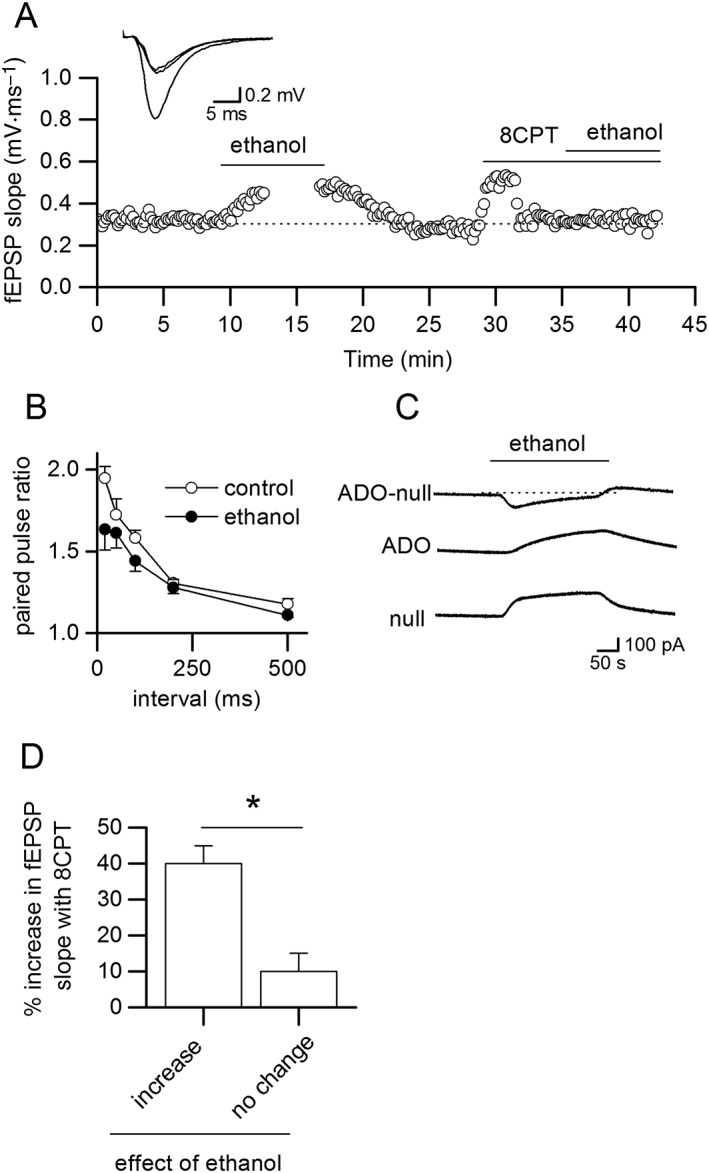
Ethanol can reduce basal A_1_ receptor continuous activation. (A) Graph plotting fEPSP slope against time for an individual slice. Ethanol (50 mM) reversibly increased fEPSP slope, which was blocked by the A_1_ receptor antagonist 8CPT (2 μM, the stimulus was reduced to return the fEPSP slope to control values before ethanol was applied). The gap in recording during the first ethanol application is for paired pulse recording. Inset, fEPSP averages in control, 50 mM ethanol and in wash. (B) The paired pulse data taken from (A) showing that ethanol reduces the paired pulse ratio at short intervals (up to 100 ms) but had little effect at longer intervals (*n* = 6). (C) Traces from an ADO biosensor with the null subtracted, ADO biosensor and null sensor. Ethanol (50 mM) induced a net downward shift in the ADO biosensor with null subtracted consistent with a fall in the extracellular concentration of adenosine. (D) Bar chart plotting the increase in fEPSP slope produced by 8CPT separated into those slices where ethanol enhanced fEPSP slope and those slices where ethanol had little effect (*n* = 9). *P< 0.05.

We also observed that in a subset of slices, ethanol could reversibly increase fEPSP slope (in nine slices, mean increase in fEPSP slope 40.1 ± 6%, Figure 3 also observed in Diao and Dunwiddie, 1996). This increase in fEPSP slope was accompanied by a significant reduction in the paired pulse ratio (for a 50 ms interval, reduced from 1.89 ± 0.05 to 1.77 ± 0.12, *P* < 0.05 one‐way ANOVA, *n* = 6, Figure 3B) and could be abolished by pre‐incubation with 8CPT (*n* = 4 slices, Figure 3A). Thus, the increase in fEPSP appears to stem from a reduction in tonic A_1_ receptor activation. If ethanol reduces the extracellular concentration of adenosine, we should be able to observe a fall in the ADO biosensor current. As previously noted, we observed a small fall in the ADO‐null current in four slices out of 40 slices (the mean drop in current was 111 ± 20 pA, equivalent to ~0.4 μM). We predicted that if the extracellular concentration of adenosine was low (little network activity), ethanol would be unable to significantly increase fEPSP slope by reducing extracellular adenosine concentration. In contrast, if the extracellular concentration of adenosine was high, then the likelihood of ethanol increasing fEPSP slope, by reducing adenosine concentration, would be increased. To test this prediction, we compared the effects of blocking A_1_ receptors (with 8CPT 2 μM) with the effects of ethanol on fEPSP slope (ethanol was applied first, then washed off, then 8CPT was applied). There was significantly (*P* < 0.05, one‐way ANOVA) greater A_1_ receptor activation in slices when ethanol markedly increased fEPSP slope compared with slices where ethanol had little or no effect on fEPSP slope (Figure 3C). This data suggest that ethanol effects are bidirectional as it can increase or decrease A_1_ receptor activation. The reason for such variability remains unclear but may contribute to the reported complex effects of adenosine on basal synaptic transmission in the hippocampus.

### Ethanol modulates adenosine release during electrographic seizure activity

**Figure 4:**
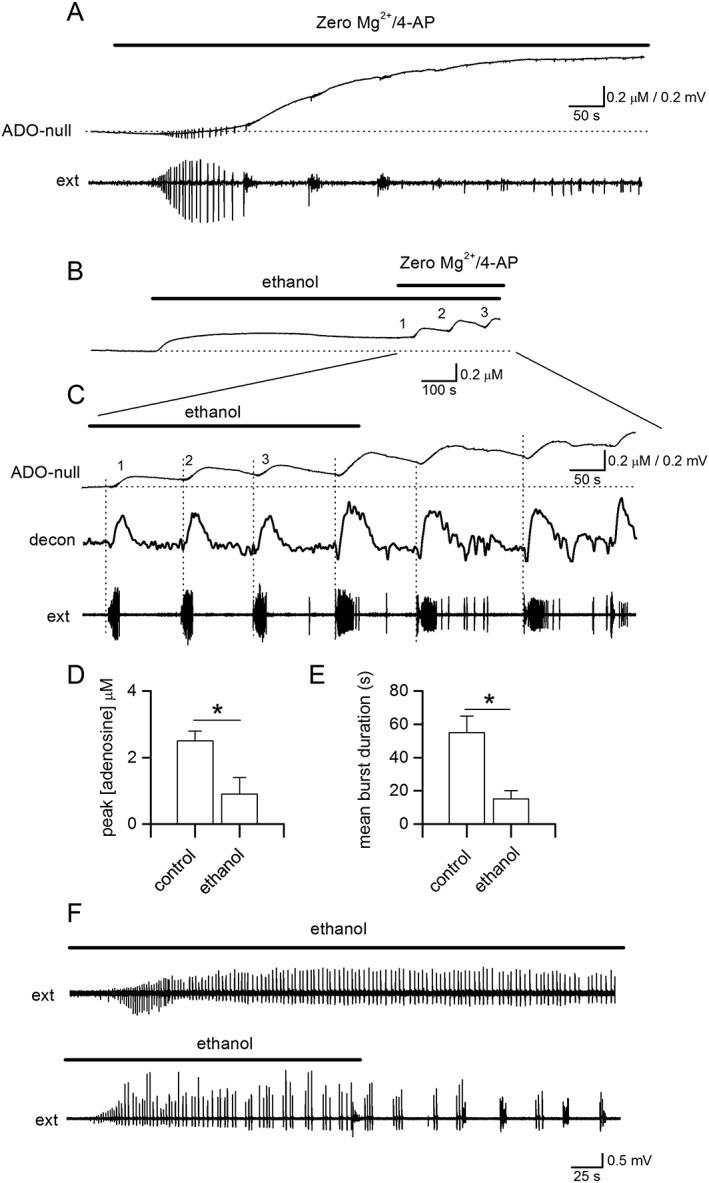
Ethanol pre‐incubation reduces adenosine release and changes seizure activity. Recordings from interleaved slices. (A) Control slice; ADO biosensor trace (null subtracted) and extracellular trace. Seizure activity induced with zero Mg^2+^ and 50 μM 4‐AP. (B) Slice was pre‐incubated in 50 mM ethanol before seizure activity. Ethanol induced an increase in the extracellular concentration of adenosine as shown by the upward shift in the baseline. (C) Expanded trace from (B) with adenosine‐release pulses deconvolved (time constant 560 s). Seizure activity increased extracellular adenosine concentration in both slices, but markedly less adenosine was released in the pre‐incubated slice (peak concentration after three bursts of activity, control 1.5 vs. 0.2 μM ethanol). This inhibition of adenosine release was partially reversed in wash with an increase in burst duration. (D) Bar‐chart summarizing peak concentrations of adenosine measured in control slices and slices incubated in ethanol (*n* = 6). (E) Bar chart summarizing mean burst duration measured in control slices and slices incubated in ethanol (*n* = 6). (F) Extracellular recordings from two interleaved slices that were pre‐incubated in 50 mM ethanol. The induced activity was continuous and not in isolated bursts until ethanol was washed out. *P<0.05.

We investigated whether ethanol modulated adenosine release during electrographic seizure activity. In slices pre‐incubated in ethanol (see Methods), there was a reduction in seizure‐induced adenosine release. Figure 4 illustrates data from two interleaved slices. Application of ethanol increased the basal extracellular concentration of adenosine (upward shift in baseline Figure 4B). Such an effect was observed in three out of six slices, with no change in baseline in the other three slices. There was no significant change in the latency to the first burst of activity in control slices and slices treated with ethanol. Significantly less adenosine was released in the pre‐incubated slice compared with the control slice (Figure 4D). In ethanol, the adenosine waveforms were of significantly (Figure 4E) shorter duration than in control, which mirrored the duration of network bursts (Figure 4C). This reduction in burst length may partly explain the fall in adenosine release. Following ethanol wash, there was a partial recovery with greater burst duration leading to more adenosine release (Figure 4C). In some slices, pre‐incubation in ethanol altered seizure activity, so it was not isolated bursts (Figures 4A, C) but was instead continual activity (Figure 4F, *n* = 4 slices). Following wash, seizure activity reverted to isolated bursts (Figure 4F).

**Figure 5:**
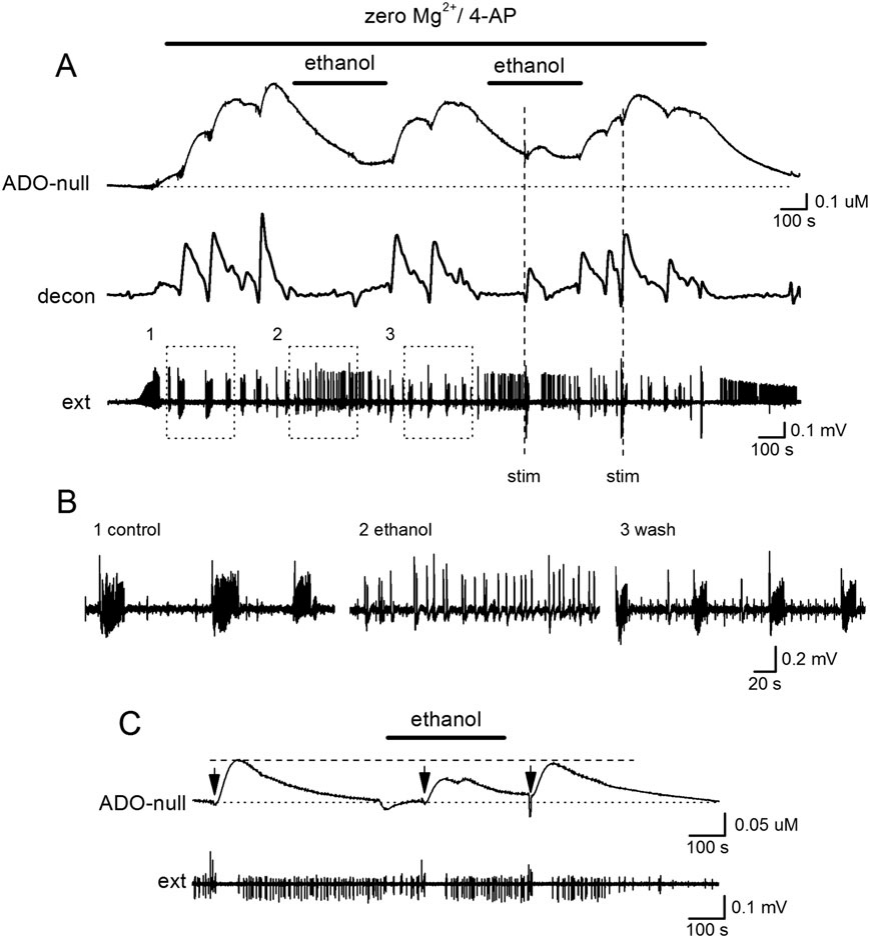
Ethanol applied during established seizures inhibits adenosine release and changes network activity. (A) Traces from an adenosine biosensor (null subtracted), data deconvolved (decon, time constant 250 s) and network activity recorded with an extracellular electrode (ext). Ethanol (50 mM) inhibited adenosine release and changed the pattern of activity. Electrical stimulation (stim) released a greater amount of adenosine once ethanol was washed out. (B) Portions of the extracellular recording from (A, dotted boxes) illustrate how ethanol changed the pattern of activity. (C) Trace from an ADO biosensor (null subtracted) with network activity (ext) from a different hippocampal slice. Electrical stimulation (50 stimuli, 20 Hz at arrows) during a period of low network activity evoked adenosine release, which was reversibly inhibited by ethanol (50 mM).

When ethanol was applied during seizure activity (see Methods), it had two clear effects (*n* = 10 slices): it inhibited the release of adenosine, resulting in a fall in the biosensor current (mean inhibition 65 ± 25% *n* = 6 slices, Figure 5A). This was especially obvious when the adenosine release pulses were separated by deconvolution (Figure 5A, decon). In most slices (four out of six), this effect was partly reversible upon wash (Figure 5A). Ethanol also changed the pattern of network activity from separated bursts into continuous short duration bursts or population spikes (Figure 5A, B) similar to the effects of blocking A_1_ receptors with a receptor antagonist (e.g. see Wall and Richardson, 2015; Lopatář *et al.,* 2015). Again, this effect was reversible in some slices. The disruption of network activity is consistent with the inhibition of activity‐dependent adenosine release and a fall in A_1_‐receptor activation.

### Ethanol inhibits electrically stimulated adenosine release

**Figure 6:**
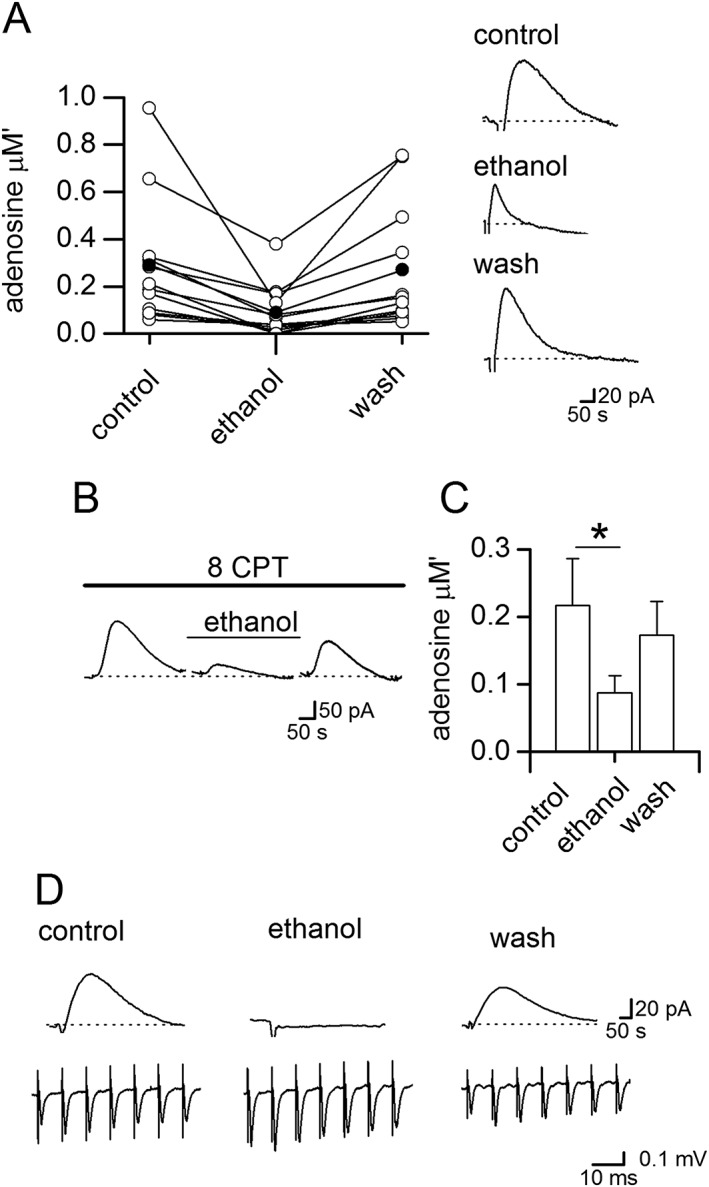
Ethanol inhibits electrically stimulated adenosine release. (A) Graph summarizing effects of 50 mM ethanol on electrically stimulated adenosine release [open circles, individual experiments; filled circles, mean data (*n* = 15)]. Inset, adenosine biosensor traces from an individual experiment in control, ethanol and following wash. (B) Stimulated adenosine release‐events recorded with an adenosine biosensor in the presence of the A_1_ receptor antagonist 8CPT. Ethanol (50 mM) still inhibited adenosine release, an effect, which was reversed in wash. (C) Bar chart summarizing data from seven recordings where ethanol (50 mM) significantly (*P < 0.05) decreased adenosine release in the presence of 8CPT. (D) fEPSPs (from the start of trains of stimuli used to evoke adenosine release) were recorded at the same time as biosensor measurements. Although ethanol reversibly abolished adenosine release‐events, the fEPSPs increased in amplitude.

The effects of ethanol are difficult to interpret; the ethanol‐induced fall in adenosine release could change network activity or conversely an ethanol‐induced change in network activity could reduce adenosine release. To dissect these mechanisms, we controlled network activity by electrically stimulating in CA1 during lulls in activity or during periods of low activity (lack of bursts) and found that ethanol still decreased adenosine release (Figure 5A, D; mean inhibition 71 ± 23%, *n* = 4). However, ethanol may still modulate any seizure activity induced by electrical stimulation, and thus, we repeated stimulation in basal conditions. Trains of electrical stimuli were delivered in CA1 (see Wall and Dale, 2013). In the majority of slices (12 out of 15 slices), ethanol (50 mM) significantly (*P* < 0.05, one‐way ANOVA) inhibited adenosine release (Figure 6A, mean inhibition 66 ± 7%, *n* = 12). Adenosine release significantly (*P* < 0.05, one‐way ANOVA) recovered in wash (93 ± 5% recovery) and was concentration‐dependent (10 mM ethanol 39 ± 8% inhibition, *n* = 4 slices). Since the pattern of network activity is controlled, ethanol must directly inhibit adenosine release.

Since ethanol can enhance basal A_1_‐receptor activation, this could inhibit activity‐dependent adenosine release (see Wall and Dale, 2013). To test this possibility, adenosine release was evoked in the presence of 8CPT, an A_1_ receptor antagonist (2 μM, Figure 6B, C). The mean inhibition by ethanol in 8CPT (60.6 ± 10%, *n* = 7) was not significantly (*P* < 0.05, unpaired *t*‐test) different from control inhibition. Thus, an increase in activation of A_1_ receptors is not a major mechanism for the inhibition of activity‐dependent adenosine release. Electrically stimulated adenosine release in the hippocampus is glutamate receptor‐dependent with both AMPA and NMDA receptors involved (Wall and Dale, 2013). Ethanol could reduce activity‐dependent adenosine release by inhibiting glutamate release. This seemed unlikely as this was not observed when we recorded fEPSPs where the inhibitory effects of ethanol were blocked by 8CPT. However, to confirm it, we recorded fEPSPs simultaneously with biosensor measurements of the stimulated adenosine release (*n* = 4). There was no clear relationship between the inhibition of adenosine release and the effects of ethanol on fEPSPs. For example in Figure 6D, although the amplitude of fEPSPs was increased in ethanol (~75%), adenosine release was abolished, an effect that was reversed upon wash. Thus, the ethanol‐mediated inhibition of activity‐dependent adenosine release appears independent of an effect on glutamate release.

### Ethanol preferentially blocks a slow component of adenosine release

**Figure 7:**
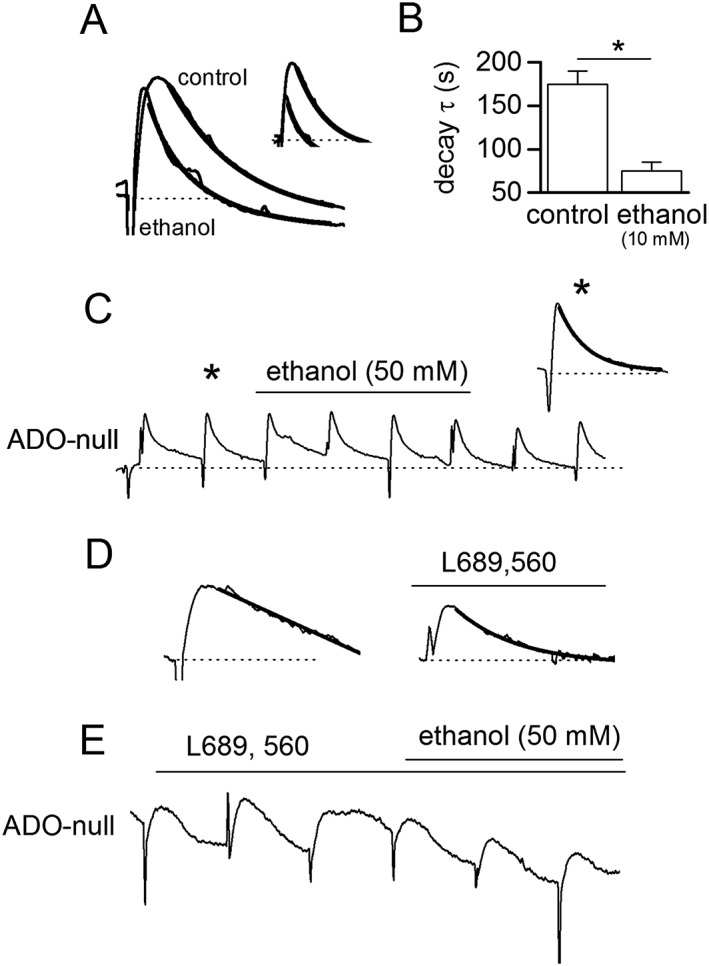
Ethanol inhibits an NMDA receptor‐dependent component of adenosine release. (A) Superimposed and normalized adenosine waveforms in control and in 10 mM ethanol. The waveform in ethanol has a faster decay than in control (decay fitted with single exponentials, τ = 220 and 69 s). Inset, waveforms from (A) superimposed but not normalized. (B) Graph summarizing the mean time constant for exponentials fitted to the decay of ADO biosensor waveforms in control and in ethanol (*n* = 5). (C) Example of an ADO biosensor trace (with null subtracted) where ethanol (50 mM) had no significant effect on stimulated‐adenosine release. Inset, expanded adenosine release event taken (*) with the decay fitted with a single exponential (τ = 62 s). (D) Adenosine waveforms in control and following application of L689560 (5 μM) to block NMDA receptors. The waveform decays are fitted with single exponentials (control τ = 320 s; L689,560 τ = 95 s). (E) Following L689,560 (5 μM) application, ethanol (50 mM) had little effect (mean reduction 7 ± 5%, no different to normal run down) on the stimulated release of adenosine.

We observed that in many biosensor recordings, the low amplitude adenosine waveform that persisted in 50 mM ethanol had a different time course to the control waveforms (e.g. see inset in Figure 6A). To examine this further, we used a lower concentration of ethanol (10 mM), which had less effect on the amplitude of the biosensor signal, so we could measure the kinetics of the adenosine‐waveform more accurately (Figure 7A). We found that that the decay of the adenosine waveforms in ethanol was significantly faster than those in control (Figure 7B). We also examined the slices where ethanol had no significant effect on adenosine release and found that in these slices, the adenosine waveforms had a significantly faster decay than in slices where ethanol produced inhibition (Figure 7C, mean time constant 75 ± 10 vs. 155 ± 20 s, *n* = 5). These data suggest that ethanol preferentially blocks a slow component of stimulated‐adenosine release. It also suggests that this component can be absent in some slices where ethanol has little or no effect on activity‐dependent adenosine release.

### NMDA receptors play a role in the actions of ethanol

We have previously shown that electrically stimulated hippocampal adenosine release is both AMPA and NMDA receptor‐dependent (Wall and Dale, 2013). Blocking NMDA receptors reduces adenosine release by on average ~70%, with little effect in some slices but complete block of release in others (Wall and Dale, 2013). These are similar to the variable effects that ethanol produces on electrically stimulated adenosine release. Furthermore, it has been reported that ethanol can block NMDA receptors (Lovinger *et al.,* 1990), and thus, this could be the mechanism of how ethanol reduces adenosine release. To investigate this further, we compared the effects of ethanol (50 mM) with the effects of blocking NMDA receptors with the antagonist L689560 (5 μM) on stimulated adenosine release in the same slices. In five slices, both ethanol and L689560 abolished electrically stimulated adenosine release (ethanol was first applied, washed and then L689560 was applied). In a further four slices, application of L689560 partially blocked stimulated adenosine release (mean inhibition 61 ± 5%), leaving a component with a faster decay (Figure 7D) that is very similar to the effects that are observed with ethanol application (Figure 7A, B). To test whether the effects of ethanol can be accounted for by the block of NMDA receptors, we attempted to occlude the effects of ethanol on stimulated adenosine release, by first blocking NMDA receptors with the antagonist L689560. In 10 slices, NMDA receptors were blocked (5 μM L689560) that partially reduced the electrically stimulated release of adenosine, and then ethanol (50 mM) was applied. In six out of 10 slices, ethanol had no significant (*P* > 0.05. one‐way ANOVA) effect on adenosine release consistent with the inhibitory effects of ethanol occurring *via* the block of NMDA receptors (Figure 7E). However, in four out of the 10 slices, ethanol still abolished the stimulated adenosine release. This suggests that ethanol can have additional effects, as well as blocking NMDA receptors, that contribute to the inhibition of adenosine release.

### Low concentrations of ethanol modify adenosine release and seizure activity

**Figure 8:**
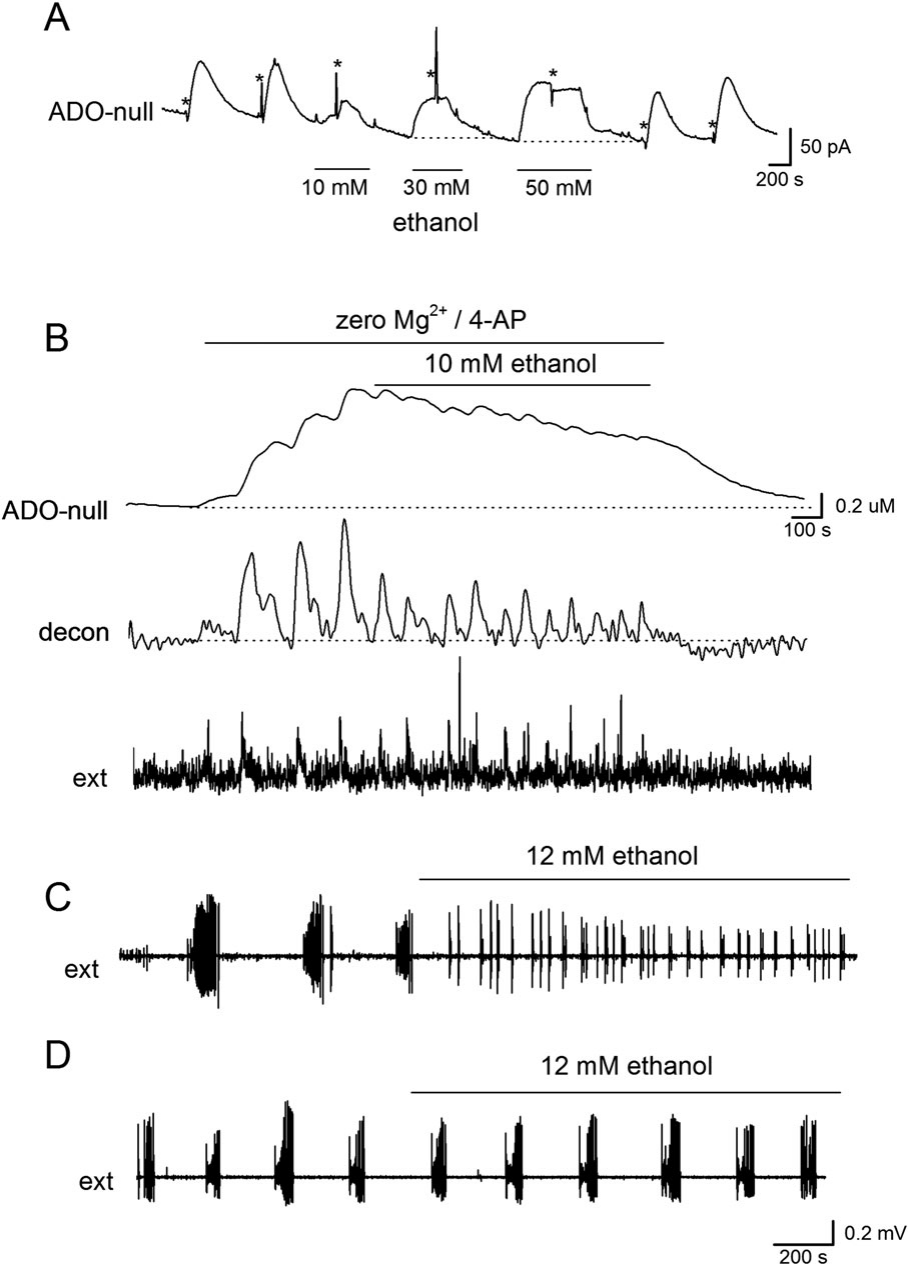
Low concentrations of ethanol reduce adenosine release and modify seizure activity. (A) Trace from an ADO biosensor (null subtracted). Adenosine release was stimulated (20 Hz 50 stimuli, at asterisks). Ethanol (10 mM) inhibited adenosine release but did not increase the baseline current. Higher concentrations abolished adenosine release but also increased the extracellular concentration of adenosine. These effects were reversible upon wash. (B) Traces are shown from an adenosine biosensor (null subtracted), these data are deconvolved (time constant 250 s), and the extracellular activity was extracted from the biosensor. Ethanol (10 mM) reduced adenosine release and the interval between bursts. (C,D) Ethanol (12 mM) either changed extracellular activity (C) or had no effect (D).

In most experiments, we have used 50 mM ethanol, which is a concentration that can be measured in the blood stream of heavy drinkers (reviewed in Harrison *et al*., 2017). We were interested in the effects that lower concentrations of ethanol could have on adenosine release. Thus, we used 10–15 mM ethanol, a concentration of ethanol found with social drinking (Harrison *et al*., 2017). At this concentration, ethanol could still inhibit stimulated adenosine release (~50%; Figures 7A and 8A, *n* = five out of 8 slices) without inducing changes in the basal concentration of adenosine (Figure 8A). We then investigated the effect of 10–15 mM ethanol on seizure activity. Pre‐incubation with ethanol (10–15 mM) significantly shortened the latency to activity onset (from 166 ± 6 to 120 ± 15 s, *P* < 0.05, unpaired *t*‐test, *n* = 6) consistent with a reduction in adenosine release suppressing activity. In some slices, ethanol also reduced activity‐dependent adenosine release (Figure 8B, mean inhibition 55 ± 15%, *n* = 4) and reduced both the interval between bursts and burst duration (Figure 8B, C). In other slices, it had little effect on either adenosine release or the network activity (Figure 8D, *n* = 4). Thus, even at low concentrations, ethanol can modulate adenosine release leading to changes in network activity.

## Discussion

Using biosensor measurements and electrophysiology, we have investigated the effects that ethanol has on adenosine release and network activity in the hippocampus. Perhaps not surprisingly, the effects of ethanol were complex and variable. There were however two clear results: (i) ethanol can alter the basal extracellular concentration of adenosine leading to changes in A_1_ receptor activation and (ii) ethanol can inhibit activity‐dependent adenosine release. The net balance between these effects could potentially supress activity, enhance activity or have no significant effect (as observed in our fEPSP recordings and that reported by Diao and Dunwiddie, 1996). These variable effects on adenosine signalling probably contribute to the inconsistent effects of ethanol that have been reported in the literature.

### Ethanol has minor effects on microelectrode biosensor properties

This is the first study to use microelectrode biosensors to directly investigate the effects of ethanol on adenosine release and its extracellular concentration. We found that ethanol had no inhibitory effect on adenosine biosensor sensitivity. This is supported by information from the enzyme database BRENDA (Placzek *et al.,* 2017), which does not list ethanol as an inhibitor of any of the adenosine‐sensing enzymes. Ethanol does induce a current on both the ADO biosensor and the null sensor, which is consistent with the direct oxidation of ethanol, with the liberation of electrons to produce the sensor current. However, the currents were small, illustrating the effectiveness of the screening layer and were linearly related to ethanol concentration and can be removed by subtraction. Thus, microelectrode biosensors are a useful tool to study the effects of ethanol on brain function.

### Ethanol modulates the background extracellular activation of adenosine receptors

Acute ethanol exposure can increase the basal activation of A_1_ receptors (reducing fEPSP slope), have no net effect or decrease basal A_1_ receptor activation (increasing fEPSP slope). These inconsistent effects on basal synaptic transmission have also been reported by Diao and Dunwiddie (1996). The enhanced A_1_ receptor activation was produced by an increase in the extracellular concentration of adenosine, which was rapid and dependent on the concentration of ethanol (threshold ~20 mM). The precise mechanism for this effect remains unclear. It does not appear to be glutamate receptor‐dependent (unlike electrically stimulated adenosine release) and does not appear to result from ENT inhibition. It has been reported that acute infusion of ethanol into the hypothalamus leads to a rapid and direct release of adenosine *in vivo* (Sharma *et al*., 2010). The fall in the increase in extracellular adenosine concentration with repeated ethanol applications suggests either rapid tolerance or depletion of the intracellular adenosine pool (Pearson *et al.,* 2001; Klyuch *et al.,* 2011). It would be interesting to see if this effect of ethanol is still observed in tissue from naïve animals.

Ethanol can also reduce the basal activation of A_1_ receptors, enhancing synaptic transmission. The mechanism for this could be a reduction in activity‐dependent adenosine release, although a clear fall in ADO biosensor baseline was not observed. In some slices, the A_1_ receptor basal activation was high, probably reflecting increased network activity. It is possible that this network activity is highly localized (around extracellular electrode), and thus, changes in adenosine concentration will not be detected (Wall and Richardson, 2015). The fall in basal A_1_ receptor activation could also result from ethanol inhibiting NTPDases (Rico *et al.,* 2008) reducing conversion of ATP to adenosine.

### Ethanol inhibits activity‐dependent adenosine release

During epileptiform activity, the amount of adenosine released into the extracellular space was reduced by ethanol. By controlling the pattern of network activity, we showed that the reduction in adenosine release can occur independently of network activity changes. Ethanol inhibits NMDA receptors (Lovinger *et al.,* 1990; Wirkner *et al.,* 1999; Möykkynen and Korpi, 2012) with 25 mM having a marked effect (Lovinger *et al.,* 1990). It has previously been shown that NMDA receptor activation in the hippocampus releases adenosine (Manzoni *et al.,* 1994; Wall and Dale, 2013), and blocking NMDA receptors markedly inhibits stimulated adenosine release (Wall and Dale, 2013). Experiments where an NMDA receptor antagonist was used to occlude the effects of ethanol on activity‐dependent adenosine release were successful in some slices but not in others. This suggests that there are additional mechanisms for reducing adenosine release. One such mechanism could be the depletion of internal adenosine stores. The increase in the basal extracellular concentration of adenosine could deplete stores of adenosine so that there is less adenosine to be released by activity. Pearson *et al*. (2001) showed that such a depletion of stores can occur during ischaemia, and Klyuch *et al*. (2011) showed that prolonged electrical stimulation depletes adenosine stores that recover when stimulation stops. During seizure activity, changes in the pattern of activity may also contribute to a reduction in adenosine release.

The effects of ethanol on adenosine signalling appear contradictory: it can enhance the basal extracellular concentration of adenosine but can also inhibit electrically stimulated adenosine release and adenosine release during seizure activity. A full explanation for this duality of ethanol effects is lacking, but it appears that the effects occur *via* separate mechanisms. It also appears that the effects on stimulate release occur with lower concentrations of ethanol than that on the basal extracellular adenosine concentration.

### Actions of ethanol on network activity during seizures

The effects of ethanol on network activity during seizures were variable and depended on when the ethanol was applied. Both high and low concentrations of ethanol could convert isolated bursts of activity into continuous activity. The loss of activity‐dependent adenosine release removes the negative feedback provided by A_1_ receptor activation promoting continuous activity. This is a similar to effects produced by A_1_ receptor antagonists. However, the other effects of ethanol will also contribute to changes in activity such as the enhancement of GABA_A_ receptor activation. The inhibition of adenosine release could potentially make ethanol pro‐convulsant, but they are offset by an increase in basal adenosine concentration, inhibition of NMDA receptors and other effects such as the enhancement of GABA effects (Harrison *et al.,* 2017). Low concentrations of ethanol, which may selectively reduce adenosine release, are more likely to be pro‐convulsant, as there is less effect on GABA and NMDA receptors. This is supported by our observations that in some slices, low concentrations of ethanol can reduce the latency to seizure activity, lengthen bursts of activity and convert isolated bursts of activity into continuous activity. However, the effects were variable with no clear effects in around half of the slices tested.

### Conclusions

Do these data on the effects of acute ethanol exposure on adenosine signalling allow us to extrapolate to human epileptic patients and ethanol drinkers and provide advice on ethanol drinking habits? It appears that low doses of ethanol can be pro‐convulsant as they inhibit adenosine activity‐dependent release and can enhance activity. It may therefore be advisable for epileptics to avoid ethanol altogether rather than having a small amount. Higher doses of ethanol are anticonvulsant, supressing activity but there is evidence that following the elimination of ethanol, seizure threshold is diminished. The results from this study have to be interpreted with caution as the tissue came from rats that were ethanol naïve, and thus, the data may best reflect the effect of ethanol on epileptic patients who are about to have their first alcoholic drink.

## Author contributions

Experimental design was by M.J.W. and M.J.E.R. Experiments were carried out by V.H. and M.J.W. Data were analysed by M.J.W. and M.J.E.R. The paper was written by M.J.W. and M.J.E.R.

## Conflict of interest

The authors declare no conflicts of interest.

## Declaration of transparency and scientific rigour

This http://onlinelibrary.wiley.com/doi/10.1111/bph.13405/abstract acknowledges that this paper adheres to the principles for transparent reporting and scientific rigour of preclinical research recommended by funding agencies, publishers and other organisations engaged with supporting research.

## References

[bph14152-bib-0001] Alexander SPH , Christopoulos A , Davenport AP , Kelly E , Marrion NV , Peters JA *et al* (2017a). The Concise Guide to PHARMACOLOGY 2017/18: G protein‐coupled receptors. Br J Pharmacol 174: S17–S129. 29055040 10.1111/bph.13878 PMC5650667

[bph14152-bib-0003] Alexander SPH , Striessnig J , Kelly E , Marrion NV , Peters JA , Faccenda E *et al* (2017b). The Concise Guide to PHARMACOLOGY 2017/18: Voltage‐gated ion channels. Br J Pharmacol 174: S160–S194. 29055033 10.1111/bph.13884 PMC5650668

[bph14152-bib-0004] Alexander SPH , Fabbro D , Kelly E , Marrion NV , Peters JA , Faccenda E *et al* (2017c). The Concise Guide to PHARMACOLOGY 2017/18: Enzymes. Br J Pharmacol 174: S272–S359. 29055034 10.1111/bph.13877 PMC5650666

[bph14152-bib-0002] Alexander SPH , Peters JA , Kelly E , Marrion NV , Faccenda E , Harding SD *et al* (2017d). The Concise Guide to PHARMACOLOGY 2017/18: Ligand‐gated ion channels. Br J Pharmacol 174: S130–S159. 29055038 10.1111/bph.13879 PMC5650660

[bph14152-bib-0005] Bettinger JC , Davies AG (2014). The role of the BK channel in ethanol response behaviours: evidence from model organism and human studies. Front Physiol 29: 346. 10.3389/fphys.2014.00346 PMC4158801 25249984

[bph14152-bib-0006] Borea PA , Gessi S , Merighi S , Varani K (2016). Adenosine as a multi‐signalling guardian angel in human diseases: when, where and how does it exert its protective effects? Trends Pharmacol Sci 37: 419–434. 26944097 10.1016/j.tips.2016.02.006

[bph14152-bib-0007] Cohen SM , Martin D , Morrisett RA , Wilson WA , Swartzwelder HS (1993). Proconvulsant and anticonvulsant properties of ethanol: studies of electrographic seizures in vitro. Brain Res 601: 80–87. 8094314 10.1016/0006-8993(93)91697-q

[bph14152-bib-0008] Curtis MJ , Bond RA , Spina D , Ahluwalia A , Alexander SP , Giembycz MA *et al* (2015). Experimental design and analysis and their reporting: new guidance for publication in BJP. Br J Pharmacol 172: 3461–3471. 26114403 10.1111/bph.12856 PMC4507152

[bph14152-bib-0109] Dale N , Frenguelli BG (2009). Release of adenosine and ATP during ischemia and epilepsy. Curr Neuropharmacol 7: 160–179. 20190959 10.2174/157015909789152146 PMC2769001

[bph14152-bib-0009] Dar MS , Mustafa SJ , Wooles WR (1983). Possible role of adenosine in the CNS effects of ethanol. Life Sci 33: 1363–1374. 6312233 10.1016/0024-3205(83)90819-6

[bph14152-bib-0010] Diao L , Dunwiddie TV (1996). Interactions between ethanol, endogenous adenosine and adenosine uptake in hippocampal brain slices. J Pharmacol Exp Ther 278: 542–546. 8768702

[bph14152-bib-0011] Dunwiddie TV , Diao L (1994). Extracellular adenosine concentration in hippocampal brain slices and the tonic inhibitory modulation of excitatory responses. J Pharmacol Exp Ther 268: 537–545. 8113965

[bph14152-bib-0012] Dunwiddie TV , Haas HL (1985). Adenosine increases synaptic facilitation in the in vitro rat hippocampus: evidence for a presynaptic site of action. J Physiol 369: 365–377. 3005559 10.1113/jphysiol.1985.sp015907 PMC1192655

[bph14152-bib-0013] Dunwiddie TV , Masino S (2001). The role and regulation of adenosine in the central nervous system. Annu Rev Neurosci 24: 31–55. 11283304 10.1146/annurev.neuro.24.1.31

[bph14152-bib-0222] During MJ , Spencer DD (1992). Adenosine: a potential mediator of seizure arrest and postictal refractoriness. Ann Neurol 32: 618–624. 1449242 10.1002/ana.410320504

[bph14152-bib-0114] Franks HM , Hagedorn H , Hensley VR , Hensley WJ , Starmer GA (1975). The effect of caffeine on human performance, alone and in combination with ethanol. Psychopharmacologia 45: 177–181. 1215448 10.1007/BF00429058

[bph14152-bib-0014] Fischer W (2005). Influence of ethanol on the threshold for electroshock‐induced seizures and electrically‐evoked hippocampal after discharges. J of Neural Trans 112: 1149–1163. 10.1007/s00702-004-0266-0 15622439

[bph14152-bib-0015] Fleuret‐Balter C , Beaugé F , Barin F , Nordmann J , Nordmann R (1983). Brain membrane disordering by administration of a single ethanol dose. Pharmacol Biochem Behav 18 (Suppl 1): 25–29. 10.1016/0091-3057(83)90142-9 6314377

[bph14152-bib-0016] Förstera B , Castro PA , Moraga‐Cid G , Aguayo LG (2016). Potentiation of gamma aminobutyric acid receptors (GABAAR) by ethanol: how are inhibitory receptors affected? Front Cell Neurosci. 10: 114 https://doi.org/10.3389/fncel.2016.00114. 27199667 10.3389/fncel.2016.00114 PMC4858537

[bph14152-bib-0017] Fredholm BB , Arslan G , Halldner L , Kull B , Schulte G , Wasserman W (2000). Structure and function of adenosine receptors and their genes. Naunyn Schmiedebergs Arch Pharmacol 362: 364–374. 11111830 10.1007/s002100000313

[bph14152-bib-0018] Frenguelli BG , Wigmore G , Llaudet E , Dale N (2007). Temporal and mechanistic dissociation of ATP and adenosine release during ischaemia in the mammalian hippocampus. J Neurochem 101: 1400–1413. 17459147 10.1111/j.1471-4159.2006.04425.x PMC1920548

[bph14152-bib-0998] Frenguelli BG , Wall MJ (2015). Combined electrophysiological and biosensor approaches to study purinergic regulation of epileptiform activity in cortical tissue. J Neurosci Methods 260: 202–214. 26381061 10.1016/j.jneumeth.2015.09.011

[bph14152-bib-0019] Gordon E , Devinsky O (2001). Alcohol and marijuana: effects on epilepsy and use by patients with epilepsy. Epilepsia 42: 1266–1272. 11737161 10.1046/j.1528-1157.2001.19301.x

[bph14152-bib-0020] Harding SD , Sharman JL , Faccenda E , Southan C , Pawson AJ , Ireland S *et al* (2018). The IUPHAR/BPS Guide to PHARMACOLOGY in 2018: updates and expansion to encompass the new guide to IMMUNOPHARMACOLOGY. Nucl Acids Res 46: D1091–D1106. 29149325 10.1093/nar/gkx1121 PMC5753190

[bph14152-bib-0921] Harrison NL , Skelly MJ , Grosserode EK , Lowes DC , Zeric T , Phister S *et al* (2017). Effects of acute alcohol on excitability in the CNS. Neuropharmacology 122: 36–45. 28479395 10.1016/j.neuropharm.2017.04.007 PMC5657304

[bph14152-bib-0021] Hendler R , Ramchandani VA , Gilman J , Hommer DW (2013). Stimulant and sedative effects of alcohol. Curr Top Behav Neurosci 13: 489–509. 21560041 10.1007/7854_2011_135

[bph14152-bib-0024] Kilkenny C , Browne W , Cuthill IC , Emerson M , Altman DG (2010). Animal research: reporting *in vivo* experiments: the ARRIVE guidelines. Br J Pharmacol 160: 1577–1579. 20649561 10.1111/j.1476-5381.2010.00872.x PMC2936830

[bph14152-bib-0025] Klyuch BP , Dale N , Wall MJ (2012). Receptor‐mediated modulation of activity‐dependent adenosine release in rat cerebellum. Neuropharmacology 62: 815–824. 21933676 10.1016/j.neuropharm.2011.09.007

[bph14152-bib-0026] Klyuch BP , Richardson MJE , Dale N , Wall MJ (2011). The dynamics of single spike‐evoked adenosine release in the cerebellum. J Physiol 589: 283–295. 21078589 10.1113/jphysiol.2010.198986 PMC3043533

[bph14152-bib-2222] Leach JP , Mohanraj R , Borland W (2012). Alcohol and drugs in epilepsy: pathophysiology, presentation, possibilities, and prevention. Epilepsia 53: 48–57. 22946721 10.1111/j.1528-1167.2012.03613.x

[bph14152-bib-0027] Llaudet E , Botting NP , Crayston JA , Dale N (2003). A three‐enzyme microelectrode sensor for detecting purine release from central nervous system. Biosens Bioelectron 18: 43–52. 12445443 10.1016/s0956-5663(02)00106-9

[bph14152-bib-0028] Lopatář J , Dale N , Frenguelli BG (2015). Pannexin‐1‐mediated ATP release from area CA3 drives mGlu5‐dependent neuronal oscillations. Neuropharmacology 93: 219–228. 25645390 10.1016/j.neuropharm.2015.01.014

[bph14152-bib-0029] Lovatt D , Xu Q , Liu W , Takano T , Smith NA , Schnermann J *et al* (2012). Neuronal adenosine release, and not astrocytic ATP release, mediates feedback inhibition of excitatory activity. Proc Natl Sci USA 109: 6265–6270. 10.1073/pnas.1120997109 PMC3341061 22421436

[bph14152-bib-0031] Lovinger DM , White G , Weight FF (1990). NMDA receptor‐mediated synaptic excitation selectively inhibited by ethanol in hippocampal slice from adult rat. J Neurosci 10: 1372–1379. 2158533 10.1523/JNEUROSCI.10-04-01372.1990 PMC6570208

[bph14152-bib-0032] Mailliard WS , Diamond I (2004). Recent advances in the neurobiology of alcoholism: the role of adenosine. Pharmacol Ther 101: 39–46. 14729391 10.1016/j.pharmthera.2003.10.002

[bph14152-bib-0034] Manzoni OJ , Manabe T , Nicoll RA (1994). Release of adenosine by activation of NMDA receptors in the hippocampus. Science 265: 2098–2101. 7916485 10.1126/science.7916485

[bph14152-bib-0035] McGrath JC , Lilley E (2015). Implementing guidelines on reporting research using animals (ARRIVE etc.): new requirements for publication in BJP. Br J Pharmacol 172: 3189–3193. 25964986 10.1111/bph.12955 PMC4500358

[bph14152-bib-0037] Möykkynen T , Korpi ER (2012). Acute effects of ethanol on glutamate receptors. Basic Clin Pharmacol Toxicol 111: 4–13. 22429661 10.1111/j.1742-7843.2012.00879.x

[bph14152-bib-0038] Nam HW , Bruner RC , Choi DS (2013). Adenosine signaling in striatal circuits and alcohol use disorders. Mol Cells 36: 195–202. 23912595 10.1007/s10059-013-0192-9 PMC3887972

[bph14152-bib-0339] Newman EA (2004). Glial modulation of synaptic transmission in the retina. Glia 47: 268–274. 15252816 10.1002/glia.20030 PMC2322937

[bph14152-bib-0839] Pankratov Y , Lalo U , Verkhratsky A , North RA (2007). Quantal release of ATP in mouse cortex. Gen Physiol 129: 257–265. 10.1085/jgp.200609693 PMC2151610 17325196

[bph14152-bib-0539] Pascual O , Casper KB , Kubera C , Zhang J , Revilla‐Sanchez R , Sul JY *et al* (2005). Astrocytic purinergic signaling coordinates synaptic networks. Science 310: 113–116. 16210541 10.1126/science.1116916

[bph14152-bib-0039] Pearson T , Nuritova F , Caldwell D , Dale N , Frenguelli BG (2001). A depletable pool of adenosine in area CA1 of the rat hippocampus. J Neurosci 21: 2298–2307. 11264305 10.1523/JNEUROSCI.21-07-02298.2001 PMC6762415

[bph14152-bib-0040] Placzek S , Schomburg I , Chang A , Jeske L , Ulbrich M , Tillack J *et al* (2017). BRENDA in 2017: new perspectives and new tools in BRENDA. Nucleic Acids Res 45 (D1): D380–D388. https://doi.org/10.1093/nar/gkw952. 27924025 10.1093/nar/gkw952 PMC5210646

[bph14152-bib-0041] Richardson MJE , Silberberg G (2008). Measurement and analysis of postsynaptic potentials using a novel voltage‐deconvolution method. J Neurophysiol 99: 1020–1031. 18046003 10.1152/jn.00942.2007

[bph14152-bib-0042] Rico EP , Rosemberg DB , Senger MR , de Bem Zrizi M , Dias RD , Souto AA *et al* (2008). Ethanol and acetaldehyde alter NTPDase and 5′‐nucleotidase from zebrafish brain membranes. Neurochem Int 52: 290–296. 17698255 10.1016/j.neuint.2007.06.034

[bph14152-bib-0043] Ruby CL , Adams CA , Knight EJ , Nam HW , Choi DS (2010). An essential role for adenosine signaling in alcohol abuse. Curr Drug Abuse Rev 3: 163–174. 21054262 10.2174/1874473711003030163 PMC3922619

[bph14152-bib-0044] Sebastião AM , Ribeiro JA (2009). Adenosine receptors and the central nervous system. Handbook Experimental Pharmacology 193: 471–534. 10.1007/978-3-540-89615-9_16 19639292

[bph14152-bib-0045] Sharma R , Egemann SC , Sahota P , Thakkar MM (2010). Effects of ethanol on extracellular levels of adenosine in the basal forebrain: an in vivo microdialysis study in freely behaving rats. Alcholism: Clinical and Experimental reaserch 34 (5): 813–818. 10.1111/j.1530-0277.2010.01153.x PMC2884072 20184564

[bph14152-bib-0046] Vizi J , Knoll ES (1976) The inhibitory effect of adenosine and related nucleotides on the release of acetylcholine 10.1016/0306-4522(76)90132-9 1004713

[bph14152-bib-0047] Wall MJ , Atterbury A , Dale N (2007). Control of basal extracellular adenosine concentration in rat cerebellum. J Physiol 582: 137–153. 17446223 10.1113/jphysiol.2007.132050 PMC2075308

[bph14152-bib-0048] Wall MJ , Dale N (2013). Neuronal transporter and astrocytic ATP exocytosis underlie activity‐dependent adenosine release in the hippocampus. J Physiol 591: 3853–3871. 23713028 10.1113/jphysiol.2013.253450 PMC3764633

[bph14152-bib-0049] Wall MJ , Richardson MJ (2015). Localized adenosine signaling provides fine‐tuned negative feedback over a wide dynamic range of neocortical network activities. J Neurophysiol 113: 871–882. 25392170 10.1152/jn.00620.2014 PMC4312871

[bph14152-bib-0050] Walter HH , Messing RO (1999). Regulation of neuronal voltage‐gated calcium channels by ethanol. J Neurochem 35: 95–101. 10.1016/s0197-0186(99)00050-9 10405992

[bph14152-bib-0051] Wirkner K , Poelchen W , Köles L , Mühlberg K , Scheibler P , Allgaier C *et al* (1999). Ethanol‐induced inhibition of NMDA receptor channels. Neurochem Int 35: 153–162. 10405999 10.1016/s0197-0186(99)00057-1

